# Behavioral Models of Tinnitus and Hyperacusis in Animals

**DOI:** 10.3389/fneur.2014.00179

**Published:** 2014-09-17

**Authors:** Sarah H. Hayes, Kelly E. Radziwon, Daniel J. Stolzberg, Richard J. Salvi

**Affiliations:** ^1^Center for Hearing and Deafness, Department of Communicative Disorders and Sciences, University at Buffalo, The State University of New York, Buffalo, NY, USA; ^2^Department of Physiology and Pharmacology, Schulich School of Medicine and Dentistry, University of Western Ontario, London, ON, Canada

**Keywords:** tinnitus, hyperacusis, lick suppression, operant conditioning, startle reflex, reaction time, behavior

## Abstract

The phantom perception of tinnitus and reduced sound-level tolerance associated with hyperacusis have a high comorbidity and can be debilitating conditions for which there are no widely accepted treatments. One factor limiting the development of treatments for tinnitus and hyperacusis is the lack of reliable animal behavioral models of these disorders. Therefore, the purpose of this review is to highlight the current animal models of tinnitus and hyperacusis, and to detail the advantages and disadvantages of each paradigm. To date, this is the first review to include models of both tinnitus and hyperacusis.

## Introduction

Subjective tinnitus refers to the perception of a sound in one or both ears, or from inside the head, in the absence of an external acoustic source (1, 2). The phantom sound of tinnitus is a serious condition affecting 10–15% of the general population, with ~1% of the population experiencing a debilitating form of chronic tinnitus that interferes with daily life (3). Hyperacusis, defined as a hypersensitivity to moderate-intensity sounds (4–7), is a condition affecting ~6% of the general population and often co-occurs with tinnitus (4). The prevalence of hyperacusis in the tinnitus population has been estimated to be as high as 80% (8), suggesting a common mechanism of dysfunction for these two perceptual disorders.

At present, the neural basis of tinnitus and hyperacusis remains elusive, and there are no widely accepted treatments or cures for individuals suffering from these conditions. However, studies in both humans and animals have led to a number of proposed neurophysiological models thought to underlie these conditions including tonotopic map reorganization, changes in spontaneous activity, or altered neural synchrony along the auditory pathway [for review, see Ref. (9)]. Rigorous testing of these hypotheses, as well as screening for potential therapeutic treatments, requires a reliable animal behavioral paradigm that not only identifies animals with tinnitus and/or hyperacusis but also allows for the use of invasive techniques, such as electrophysiological recordings from the brain and neuroanatomy, which are inappropriate for use in human patients.

In order to identify and investigate potential underlying mechanisms of tinnitus and hyperacusis, a number of animal models have been developed (10–15). Since an animal cannot directly communicate its subjective experiences, behavioral paradigms that extrapolate an animal’s perception based on changes in behavioral performance have been devised to indicate whether an animal is experiencing tinnitus and/or hyperacusis. Such paradigms have utilized a number of behavioral training techniques including lick or lever pressing suppression (10–14), two-choice operant conditioning (16–18), and reflex modification (15). Ultimately, any behavioral model of hyperacusis or tinnitus should closely mirror what we know about these disorders in the human population. When evaluating animal models of tinnitus and hyperacusis, a number of important factors should be considered, including whether the method, time-course, and variability of tinnitus or hyperacusis induction, as well as any measures of pitch or loudness, are consistent with evidence from human tinnitus/hyperacusis patients. Furthermore, behavioral paradigms should be resistant to confounding influences, such as hearing loss, that often accompany noise or drug-induced tinnitus/hyperacusis.

Thus, the goal of this review will be to evaluate current animal behavioral models of tinnitus and hyperacusis with a focus on the most widely used and newest paradigms in the literature. For each paradigm, a brief summary will be provided as well as a discussion of the paradigm’s major advantages and disadvantages. Important factors such as consistency with the human condition and resilience to the secondary effects of drug or noise exposure will also be discussed. Although previous reviews have thoroughly evaluated many of the proposed animal models of tinnitus (19–25), to our knowledge, this review will be the first to incorporate evaluations of animal models of hyperacusis, as well as models of tinnitus, which is necessary given the frequent co-occurrence of these two disorders.

## Human Studies of Tinnitus

In order to evaluate animal models of tinnitus, it is important to have an understanding of the key characteristics of tinnitus in the human population. Much of what we know about the features of tinnitus comes from subjective descriptions by tinnitus patients. Studies in which tinnitus is induced in individuals following exposure to loud sound or ototoxic agents, as well as studies of individuals with long-standing tinnitus, provide us with information regarding tinnitus pitch, intensity, time course for onset following exposure, and variability of induction.

**Figure 1:**
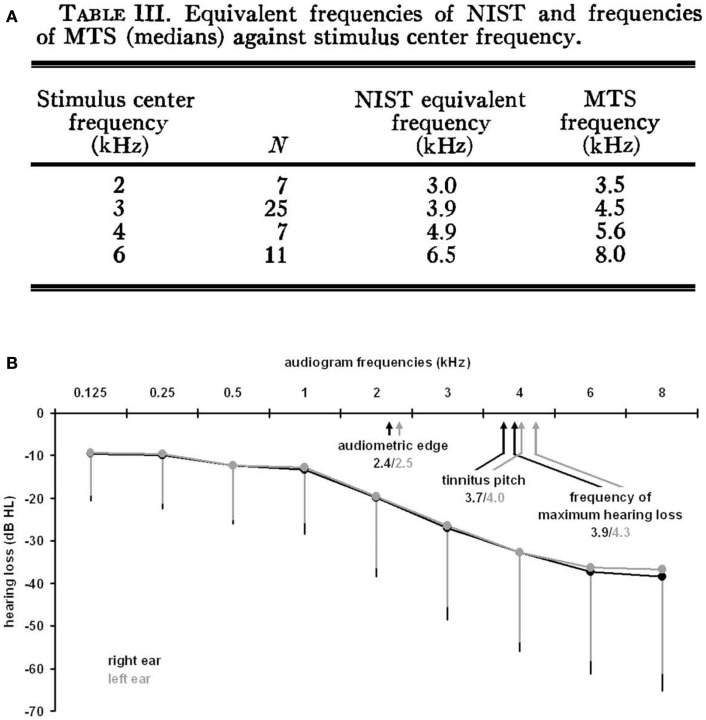
**Measurements of tinnitus pitch in human subjects with acute noise-induced tinnitus or long-standing tinnitus**. **(A)** Following acute noise exposure, tinnitus pitch has been found to occur above the frequency of the noise exposure close to the region of maximal hearing loss generated by the noise exposure [from Ref. (29) with permission; NIST, noise-induced short duration tinnitus; MTS, maximum threshold shift]. **(B)** Similarly, tinnitus pitch has been matched to frequencies in the region of maximum hearing loss for individuals with long-standing tinnitus [from Ref. (32)].

Measurements of tinnitus pitch are commonly conducted using pitch matching techniques in which individuals are presented with tones of varying frequency and asked to select the tone that is closest in pitch to their tinnitus [for a methodological review, see Ref. (26)]. A number of studies have demonstrated a link between the pitch of tinnitus and the configuration of an individual’s hearing loss. Tinnitus pitch measurements have been made in individuals immediately following exposure to loud sounds (27–29) and in individuals with long-standing tinnitus (30–33). Following acute exposure to loud sound, the pitch of tinnitus was found to occur above the frequency of the noise exposure, either in the high-frequency edge of a sharply localized hearing loss (27) or close to the region of maximum hearing loss produced by the noise exposure (28, 29) (Figure 1A). Similarly, in individuals with long-standing hearing loss and tinnitus, the tinnitus pitch was matched either to the frequencies at the edge of the hearing loss (31), or to the frequency region of maximal hearing loss (32) (Figure 1B). Tinnitus pitch matching measures have also been completed in individuals exposed to sodium salicylate, a drug known to reliably induce temporary hearing loss and tinnitus in humans and animals when taken at high doses [for review, see Ref. (34, 35)]. Although some studies have matched the pitch of salicylate-induced tinnitus and hearing loss across a broad range of frequencies, salicylate-induced tinnitus and hearing loss are both most commonly reported to occur at the high frequencies (34). In general, the pitch of tinnitus resulting from acute noise exposure or long-standing hearing loss commonly occurs within the region of hearing loss and above the frequency of the noise exposure used to induce hearing loss (36). However, measurements of tinnitus pitch are complicated by findings that tinnitus pitch also depends on the etiology of the tinnitus (37) and that tinnitus pitch tends to be lower in frequency for individuals with normal audiometric profiles, i.e., normal thresholds at 250–8000 Hz (30).

Loudness matching has also been conducted on individuals experiencing tinnitus following exposure to loud sounds or sodium salicylate, as well as in individuals with long-standing hearing loss and tinnitus. The median intensity of tinnitus resulting from acute exposure to loud noise was found to be 9 dB SL, whereas the tinnitus resulting from sodium salicylate was matched at 5–15 dB SL (29, 34). In individuals with long-standing tinnitus, the intensity reported by individuals ranges from 7.1 to 18.9 dB SL (31). However, loudness recruitment, steeper than normal loudness growth functions found in patients with hearing loss (5), can contaminate tinnitus-loudness matches by underestimating the perceived loudness of tinnitus (26).

Additional factors to consider regarding tinnitus in the human population are the time course for onset following exposure to noise or sodium salicylate and the variability of its induction. For individuals exposed briefly to loud noise, tinnitus onset is reported to occur immediately following the exposure (28, 29). Given the short duration of noise exposure in some of these studies (5 min), tinnitus was reported to last for nearly 15 min following the exposure (29). However, following exposure to a high dose of sodium salicylate, tinnitus onset occurs between 1 and 3 h post exposure and typically dissipates within 1–2 days following the exposure (34). Despite the differences in tinnitus onset for acute noise exposure and salicylate, both methods result in variability in the induction of tinnitus. Following acute noise or salicylate exposure, not all individuals develop tinnitus (28, 29, 34). Interestingly, some individuals with a preexisting hearing loss exposed to high concentrations of salicylate failed to experience tinnitus, suggesting an individual variability for susceptibility to salicylate-induced tinnitus in this population (34). Thus, tinnitus pitch, intensity, onset, and variability in the human population are important factors to consider when evaluating animal models of tinnitus.

## Animal Models of Tinnitus

### Jastreboff

The first behavioral model of tinnitus in animals was developed by Jastreboff et al. (10). In this *conditioned lick-suppression paradigm*, rats were trained to lick for water during periods in which a steady background noise was present, and to suppress their licking during brief periods of silence (conditioned stimulus), which were followed by a foot-shock (unconditioned stimulus). In the initial study, tinnitus was induced using an injection of sodium salicylate following training. During the testing phase, the foot-shock was turned off resulting in the eventual extinction of the lick-suppression behavior. The rate of extinction was assessed over multiple test days and was used as an indicator for the presence of tinnitus; specifically, animals given the tinnitus inducer salicylate began licking during the silent intervals earlier than animals given saline presumably because they heard their tinnitus during the quiet intervals. The rapid extinction of the lick-suppression behavior in the salicylate-treated rats was interpreted as the presence of tinnitus because animals with tinnitus do not experience silence and were expected to behave as if a sound is being presented.

A series of important controls were performed using this paradigm to ensure that the observed behavior was representative of tinnitus and not another confounding factor associated with salicylate administration. First, to demonstrate that the observed results were auditory-specific, a light stimulus was used in place of the background sound and animals were trained to suppress licking when the light was turned off. Salicylate administration following training with the light stimulus had no effect on the behavior, indicating that the effects of salicylate are auditory-specific. The effects of salicylate on thirst and motivation were also controlled by administering salicylate during training as well as during testing. Rats given salicylate during training associated their tinnitus perceived during the silent intervals with the foot-shock and suppressed their licking during the silent (tinnitus) intervals during testing. Salicylate administration did not result in a general effect on drinking behavior since rats administered salicylate during training *decreased* their licking during silent intervals whereas rats administered salicylate after training *increased* their licking during silent intervals. Furthermore, hearing loss as a confounding factor was also investigated by decreasing the intensity of the background sound, which did not affect the behavior.

The conditioned lick-suppression paradigm offers a number of advantages including its relatively short training time and the observation that it is not affected by confounding factors related to tinnitus induction such as hearing loss and non-auditory effects of salicylate. However, this lick-suppression paradigm is not useful for long-term studies of tinnitus because the behavior extinguishes. Since the animals are tested in extinction over a period of several days, they no longer remain under stimulus control when the shock is turned off. Additionally, the paradigm requires comparison of groups of animals (tinnitus versus control) and has not been used for assessing the presence of tinnitus in individual animals.

### Bauer and Brozoski

As mentioned above, one limitation of the Jastreboff lick-suppression paradigm was its inability to test for long-term tinnitus in rodents. Motivated by the need to test pharmaceuticals for treating tinnitus, Bauer and Brozoski developed an aversive conditioning behavioral paradigm derived from the Jastreboff model in order to provide long-term quantitative and qualitative assessment of tinnitus perception in rats (11).

Similar to the Jastreboff model, Bauer and Brozoski developed a shock-avoidance paradigm in which rats were trained to discriminate sound (white noise or tones of various frequencies and intensities) from silence (0 dB SPL). Initially, the rat’s behavior was shaped to frequently press a lever while a white noise or a tone was presented. This behavior was reinforced by a variable interval of reinforcement with a food pellet. One minute silent periods interrupted the white noise and were followed by a brief foot-shock if the lever was pressed. This procedure quickly trained the rat to avoid pressing the lever only during the silent periods. Following initial training, the foot-shock was turned on infrequently, occurring approximately once per week per rat. A lever suppression ratio, $R=\frac{B}{\left(A+B\right)},$ was used to quantify whether or not the number of lever presses during the current 1 min period, *A*, differed from the immediately preceding period, *B*. A value of *R* = 0.0 indicated complete suppression of lever pressing (i.e., rats reported no sound was present), whereas a value of *R * = 0.5 indicated no suppression of lever pressing compared to the previous white noise stimulus (i.e., a sound was present).

Behavioral performance on this task has been observed following chronic exposure to either sodium salicylate in drinking water (11) or acute unilateral noise trauma (38). In the initial study using sodium salicylate, blood-serum levels of salicylate were similar to those measured in humans with salicylate-induced tinnitus. Rats treated with salicylate demonstrated no difference in lever pressing during silent intervals compared to control animals, but did demonstrate higher *R* values (more lever pressing) to tone stimuli, with the maximum increase in *R* value occurring with the 15-kHz tonal stimulus. This was interpreted to represent the presence of tinnitus in the salicylate-treated subjects, as a maximal interaction was expected to occur at tonal frequencies that most closely resembled the tinnitus frequency. The explanation for the behavioral shift to tonal stimuli was that the salicylate-treated subjects heard the tones differently than control subjects and perceived the tones as more noise-like due to their tinnitus. In other words, the tinnitus percept was expected to interact with the perception of the externally presented tonal stimuli to produce a “noisy” percept if the tones were similar to the tinnitus pitch. Since the animals were trained to press during noise stimuli, the animals were expected to press more often when the test tones interacted with their tinnitus to create this noise-like percept. The tinnitus-like behavior was reversed in the experimental group soon after treatment with salicylate was stopped.

In a subsequent study (38), a 1- or 2-h unilateral traumatizing noise exposure centered at 16 kHz was used to induce tinnitus in rats trained on this behavioral paradigm. Following the 1-h noise exposure, the maximum shift in *R* value occurred during testing sessions where the 20-kHz test tone stimulus was presented. However, unlike the previous study in which salicylate-induced *increases* in *R* value were interpreted as the evidence of tinnitus, unilateral noise exposure resulted in a significant *decrease* in *R* value for tonal stimuli which was interpreted as the presence of tinnitus. The reduction in *R* value in the noise-exposed animals was reported for up to 17 months following noise exposure, i.e., persistent tinnitus.

Importantly, in the same study, the possible confounding effects of hearing loss were controlled by including an additional experimental group, which were not noise-exposed but were outfitted with foam earplugs fixed in the ear canal with cyanoacrylate. The suppression ratio, *R*, of this group was unaffected by the ~40 dB conductive hearing loss due to the earplug. This is strong evidence that the behavioral paradigm is robust to a moderate unilateral conductive hearing loss; however, this does not exclude the possibility that unilateral sensori-neural hearing loss, and the subsequent loudness recruitment, may be a confounding factor.

There are a few notable strengths of this behavioral paradigm for the assessment of tinnitus in rats including the ability to test subjects over long periods of time, its resilience to unilateral conductive hearing loss, ability to determine tinnitus pitch, and the rigor through which the paradigm has been tested by its creators. One limitation of the paradigm is that the results are presented as a mean of all animals performing within a group. While this approach may be effective and appropriate for testing the viability of various pharmaceuticals using group statistics, it is less effective in assessing tinnitus in an individual subject, and the time course of tinnitus onset. Another disadvantage of the paradigm is that since only one tone frequency is presented during each session, many testing sessions are required to determine the pitch of tinnitus. Additionally, using this paradigm, tinnitus-like behavior does not appear until weeks following exposure to unilateral noise trauma, a result at odds with the human literature in which tinnitus onset typically occurs immediately following exposure to intense noise (27–29). Furthermore, there is little evidence in the human literature indicating that tinnitus interferes with the perception of external tones as suggested by the authors. Indeed, external sounds ~5–15 dB above thresholds may be quite effective in suppressing tinnitus (26).

### Heffner

In an attempt to improve upon the Jastreboff lick-suppression paradigm, Heffner and Harrington (12) trained hamsters in a *conditioned suppression/avoidance procedure* to drink in the presence of a broad-band noise or various tones, and to stop drinking in the absence of these sounds (silence) to avoid a shock. Although the lick-suppression methods were similar, two crucial differences exist between the Jastreboff and Heffner paradigms. For one, the animals in the Heffner conditioned suppression paradigm underwent extensive training in the hopes of testing individual animals for tinnitus. Jastreboff, on the other hand, took less time to train each animal but could only assess groups of animals for tinnitus. Another key difference between the two paradigms is that the shock was avoidable in the Heffner conditioned suppression paradigm whereas the electric shock was unavoidable in the Jastreboff paradigm. Despite these differences, the hypothesis for both procedures remained the same. Namely, when the shock was turned off during testing, animals with noise-induced (12) or salicylate-induced (10) tinnitus were hypothesized to extinguish faster than animals without tinnitus, because animals with tinnitus no longer experience silence, and therefore, should not know when to suppress their licking.

Although tinnitus could be assessed in individual animals, the major drawback of the Heffner conditioned suppression paradigm was that a significant overlap in performance existed between the control and noise-exposed animals (25). Moreover, since this paradigm used extinction as its behavioral measure of tinnitus, this paradigm cannot be used to detect chronic tinnitus (21). For these reasons, Heffner and colleagues developed another tinnitus paradigm using a *two-choice sound localization procedure*.

During the sound localization procedure, animals were trained to lateralize sounds by responding to the right side of a test box for sounds coming from a speaker on the right side and to respond to the left side of a test box for sounds coming from the left side (16, 39). Animals were given water reward for correct responses and were shocked for incorrect responses. Importantly, silent trials, or probes, were interspersed on ~24% of trials, which were neither reinforced nor punished but the animals were forced to choose a side. The side preference during silent trials was determined for each animal prior to noise exposure.

Tinnitus was induced by sound-exposing the ear opposite each animal’s side preference during silent trials (16). For instance, an animal with a right-side bias for silent trials during training would be given a left ear sound exposure. Accordingly, animals with tinnitus should switch their side preference during silent trials from the side preferred prior to noise exposure (the right side in the above example) to the opposite, noise-exposed side (the left side in the above example) because the animals now hear a phantom sound on that exposed side. In accordance with their hypothesis, the researchers found that hamsters (16) and rats (39) will shift their side preference on silent trials to the noise-exposed, previously non-preferred ear, suggesting that they perceive a phantom sound in that ear. As an important control condition, simply plugging one ear and producing a conductive hearing loss does not result in a shift in behavior on silent trials. However, the key assumption of this paradigm is that exposing one ear to loud sound will always induce tinnitus lateralized to that ear and never produce bilateral tinnitus or tinnitus in the opposite ear. Therefore, a major drawback of this paradigm is that it cannot be used to test drug-induced tinnitus or binaural noise exposures that would likely induce bilateral tinnitus.

Yet, the advantage of using a two-choice paradigm to detect tinnitus is that animals with tinnitus make a *qualitatively* different response than animals without tinnitus, whereas animals with tinnitus in lick-suppression paradigms lick more or less than animals without tinnitus, resulting in *quantitative* differences between the two groups that may be the result of other factors, such as hearing loss, that accompany drug or noise-induced tinnitus (12). In other words, animals perceiving tinnitus in a two-choice paradigm go to a different side, or press a different lever, than animals without tinnitus, whereas animals perceiving tinnitus in suppression paradigms lick more or less than animals without tinnitus but still perform the same licking behavior in both cases. Since tinnitus animals in two-choice tasks make a qualitatively different response than non-tinnitus animals, the behavior in two-choice experiments is more resistant to changes in motivation, stress, hearing loss, or hyperacusis that frequently co-occur with drug or noise-induced tinnitus (12, 17).

### Rüttiger

Rüttiger and colleagues have developed a water-reinforced conditioned avoidance paradigm for rats with the goal of limiting the need for long periods of water deprivation as well as unavoidable shock (13). Animals are trained to shuttle between two water spouts during the presentation of a 70-dB SPL white noise background sound in order to receive a reward of 3% sugar water. During silent periods, however, the animals receive a mild foot-shock if they access the water spouts. The animals are trained over a period of weeks until their responses to the water spouts during silent periods are sufficiently suppressed compared to the responses during white noise background sound presentation. Importantly, a variable reinforcement rate is introduced during the final stages of training in order to reduce extinction of the responses, given that both the reward (sugar water) and foot-shock are turned off during the testing phase. Animals with tinnitus are expected to increase their responses at the water spouts during silent periods indicating that they are experiencing a phantom sound. This paradigm has been used to determine the presence of both salicylate and noise-induced tinnitus (13, 40). It has also been used to determine the intensity of tinnitus by comparing the response rate of rats treated with salicylate to that of animals in which the background sound was presented at varying intensities, and was estimated to be ~30 dB SPL.

This paradigm has a number of advantages. As mentioned previously, it does not require long-term deprivation of water or presentation of an unavoidable foot-shock. It can be used to test individual animals for tinnitus by injecting saline, as a control, or salicylate in the same animals on different days and comparing behavioral performance. However, although trained animals are reported to go 6–8 months without training and still perform the task to criterion, it cannot be used to test animals repeatedly for tinnitus over long durations due to extinction of the response when reward/punishment is turned off during testing and when persistent tinnitus is present following noise exposure. Furthermore, although the paradigm has been used to determine the intensity of tinnitus, it has not been used to determine the frequency of tinnitus.

### Lobarinas

Lobarinas and colleagues introduced a schedule-induced polydipsia avoidance conditioning (SIP-AC) paradigm to assess rats for salicylate-induced tinnitus (14). This paradigm differed from the previously mentioned lick-suppression paradigms in two ways. For one, the animals were not water restricted, but were food restricted and trained to lick a water spout while waiting for a food pellet to drop into a trough. In the SIP-AC paradigm, animals would receive one food pellet per minute and would consume water, even though they were not water deprived, while waiting for another pellet to drop. This behavior is referred to as polydipsia because the animals drank the water even though they were not water deprived (14). Second, unlike the other lick-suppression paradigms where the animals were trained to stop licking in the absence of sound (quiet), animals in the SIP-AC paradigm were trained to stop licking in the presence of sounds. In other words, animals could lick for water in quiet but were shocked for licking during sound trials. Therefore, if animals experience tinnitus in the SIP-AC paradigm, they should cease licking during quiet trials because they now hear a sound. The benefit of this procedure is that the shock never has to be turned off because an animal with tinnitus should not lick during sound or quiet trials, and therefore, would not get incorrectly shocked. Since the shock is never turned off, extinction is not a problem with this paradigm; therefore, it can potentially be used to measure chronic tinnitus.

However, like all of the previous lick-suppression paradigms, SIP-AC is not robust to changes in motivation or hearing loss that accompany salicylate and/or noise-induced tinnitus (23). For instance, an animal given a large dose of salicylate might become sick and less motivated to drink overall regardless of whether it has tinnitus or not. Similarly, an animal with a substantial drug or noise-induced hearing loss might mistakenly lick during a sound trial, perceiving it as a quiet trial, and receive a shock. Since the animal is not water deprived, the animal might stop licking altogether to avoid the shock. Therefore, an animal with hearing loss could conceivably test positive for tinnitus (23).

### Sederholm and Swedberg

Recently, Sederholm and Swedberg (17) trained rats in a *two-choice operant conditioning procedure* to identify rats with salicylate and noise-induced tinnitus. For this procedure, each rat was trained to press a “tone” lever when it heard a tone and to press a “0 Hz” (silence) lever when no sound was presented. Correct responses were rewarded with food and incorrect responses resulted in a reset of the fixed ratio requirement (20 additional lever presses) on the appropriate lever. After the animals were trained to criterion, the animals were tested following salicylate administration or intense noise exposure in a quiet chamber where their lever presses to the “tone” lever were counted (17). Animals with tinnitus should have a greater number of “tone” lever presses than “0 Hz” lever presses during testing even though no sound is presented.

**Figure 2:**
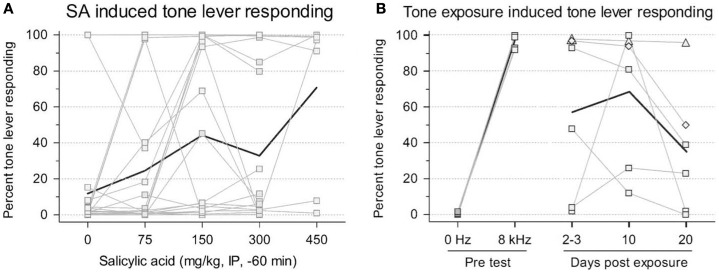
**Rats trained in a two-choice operant conditioning paradigm to press a “tone” lever when tones were presented or a “0 Hz” (silence) lever during periods in which no sound was presented in order to receive a food reward**. Changes in tone lever pressing during silent intervals were observed following exposure to salicylate **(A)** or unilateral acoustic trauma **(B)**. Thin lines represent individual animal data while bold lines depict group data. An increase in tone lever pressing during silence was used to indicate the presence of tinnitus following exposure [from Ref. (17), with permission].

The benefits of this procedure are that no shock is required to train the animals; and, given that this is a two-choice paradigm, it is more resistant to confounding factors in tinnitus induction, such as hearing loss, hyperacusis, motor impairment, and loss of motivation, since the animals make a qualitatively different response when they experience tinnitus (12, 17). However, this procedure requires extensive training (2–3 months) and the authors could only train their animals to criterion using high stimulus levels (55–65 dB SL) that are likely much higher than the perceived tinnitus intensity. Furthermore, since the rats were always reinforced for responding during testing, it is not clear how long the animals remain under stimulus control. For instance, after several testing sessions, it is possible that the animals would randomly press either lever since both levers result in food reward, making this paradigm problematic for studies of chronic tinnitus (17). In addition, lever pressing during testing appears highly variable across animals making tinnitus assessment difficult (Figures 2A,B). However, despite these criticisms, this is a new tinnitus paradigm that, with more testing, might prove useful in future tinnitus studies.

### Stolzberg

A central goal of tinnitus research is to identify a putative neurophysiological correlate of tinnitus perception. Major advances have been made toward this objective in electrophysiological studies in human tinnitus patients (41). While many reports exist on neurophysiological changes following induction of tinnitus in animal models, only a small subset have done so with behavioral confirmation of tinnitus in alert animals without the influence of anesthesia [e.g., Ref. (42)]. In an attempt to overcome this issue, Stolzberg and colleagues developed a novel appetitive *two-alternative forced choice* assay, which is better suited to investigate possible neurophysiological correlates of tinnitus in an animal model by allowing neural activity to be recorded while the animal is actively exhibiting tinnitus-like behavior (18).

**Figure 3:**
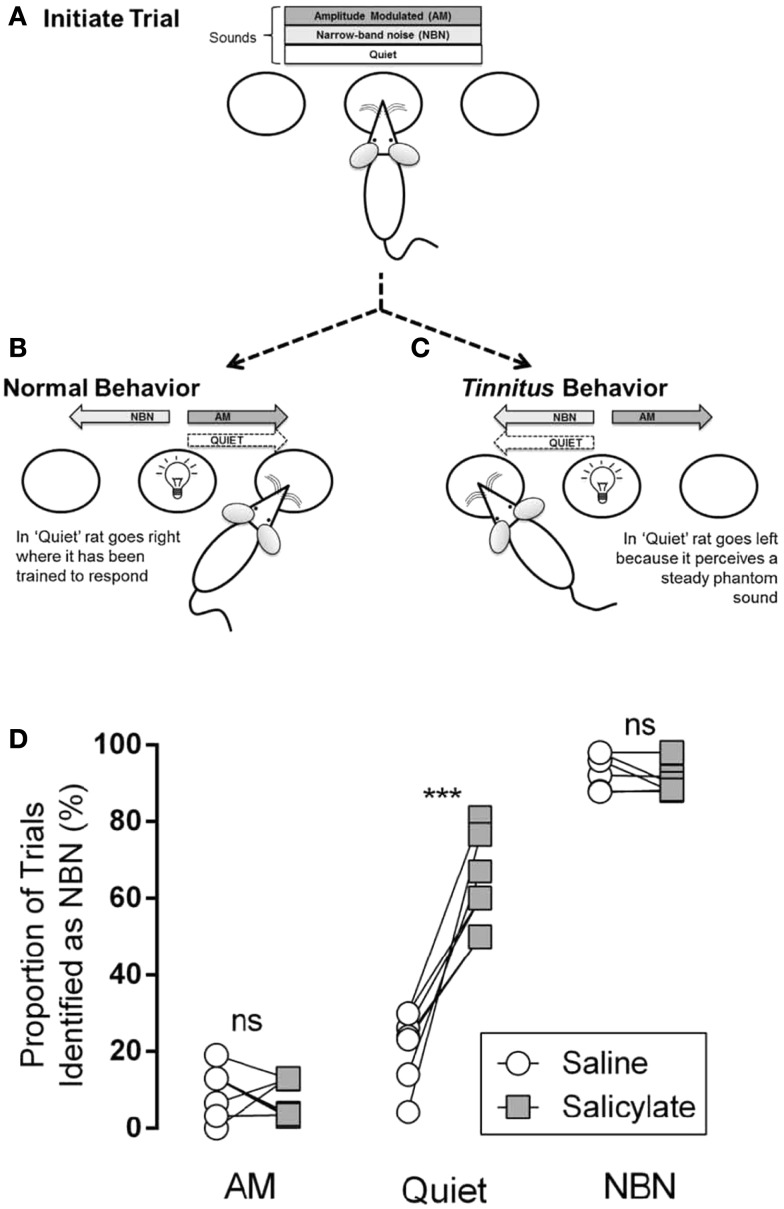
**Illustration of behavioral tinnitus assay reported in Ref. (18)**. **(A)** One of the three conditions (AM, NBN, Quiet) is present. The rat self-initiates a trial by nose-poking in the center port. **(B)** After a variable delay, a light cues the rat to respond to a feeder trough located to the left or right of the center port. The NBN condition is paired with the left feeder trough. AM and Quiet conditions are paired with the right feeder trough. **(C)** Following induction of tinnitus, if a rat hears a steady phantom sound it should respond to the left feeder during the Quiet condition while still correctly identifying AM and NBN conditions. **(D)** Comparison of performance of rats (*n* = 7; each circle–square pair represents one rat) between saline and salicylate treatments. Following saline treatment AM and Quiet conditions were infrequently misidentified as NBN, whereas NBN was identified correctly. Following salicylate treatment Quiet conditions were significantly more likely to be misidentified as NBN, indicating that salicylate induced a phantom sound perception (ns = not significant, *p* < 0.001) [from Ref. (18), with permission].

Stolzberg and colleagues trained rats to access a left feeder port in the presence of a steady, unmodulated narrow-band noise (NBN; 1/8th octave band-pass noise with a frequency center selected randomly across trials), and to access a right feeder port in the presence of a sinusoidally amplitude modulated noise (AM; broad-band noise with amplitude modulated 100% at 5 Hz) or silence (Quiet) (Figure 3B). One of the three acoustic conditions (NBN, AM, Quiet) was continuously present in the testing chamber at the start of each trial. Rats initiated a trial by nose-poking into a center port (Figure 3A) and maintained this position for a randomized period of 4–8 s until a light cued them (“go cue”) to respond to a feeder port based on the acoustic condition. Correct responses were reinforced with a food pellet and incorrect responses resulted in a “time-out” in which the rat was unable to initiate a new trial. During training, the reinforcement rate was reduced from 100 to 70% in order to minimize extinction of the learned behavior, and the percentage of trial types was divided evenly between the two feeders (NBN at 50%; AM at 30%; Quiet at 20%).

On testing days, in which rats received either an injection of saline or a high dose of salicylate to induce tinnitus, Quiet trials were neither reinforced nor punished. Tinnitus-like behavior was indicated by the rat shifting its response during Quiet trials from the right feeder associated with AM and Quiet during training, to the left feeder associated with the steady NBN (Figure 3C). Following treatment with salicylate, rats incorrectly identified the Quiet condition as a NBN significantly more than baseline or saline, indicating the presence of a steady NBN-like phantom sound (i.e., tinnitus) during Quiet trials (Figure 3D). Importantly, this change in behavior during Quiet trials was only observed following injection of salicylate and not following injection with saline. Furthermore, the rats still correctly identified AM and NBN stimuli suggesting that they were not performing randomly (Figure 3D).

This novel tinnitus behavioral assay has some distinct advantages over other tinnitus paradigms, including the ability to identify tinnitus-like behavior in individual animals and the specific design for simultaneous acquisition of electrophysiological data. During the 4–8 s period in which the animals hold their head in the center port to initiate a trial, neural activity from chronically implanted electrodes can be recorded with minimal artifact when the animal is largely immobile with its head in a fixed position in the sound field. In addition, this paradigm is very robust to the secondary effects of salicylate-induced tinnitus, such as hearing loss, hyperacusis, and hyper-reactivity, because the rats still maintain correct performance on AM and NBN stimuli so it is clear that the animals are under stimulus control. Furthermore, as in the other two-choice paradigms (16, 17), animals with tinnitus make a *qualitatively* different response than animals without tinnitus, unlike in the suppression paradigms where it is difficult to differentiate tinnitus from hearing loss, stress, or other factors associated with drug or noise exposure (12). However, it is uncertain if the paradigm in its present form is appropriate for assessing chronic tinnitus. Additionally, this paradigm does not provide information regarding tinnitus pitch or loudness.

### Turner (gap pre-pulse inhibition)

In 2006, Turner and colleagues introduced a novel tinnitus behavioral paradigm, referred to as gap pre-pulse inhibition of the acoustic startle reflex (GPIAS), which utilizes an animal’s motoric response (startle reflex) to a sudden loud sound (startle stimulus) that is recorded by a motion sensitive transducer (15). Presentation of the acoustic startle stimulus evokes a robust acoustic startle reflex (ASR); however, this reflex can be suppressed by insertion of a short duration silent gap in a continuous background sound just prior to the startle-eliciting stimulus (15, 43). In most studies, the ratio between the startle amplitude during trials in which the startle stimulus is presented alone (no-gap trials) and trials in which a gap is presented prior to the startle-eliciting stimulus (gap trials) is calculated as the GPIAS ratio. This ratio is used as an indicator of the effectiveness of the silent gap to inhibit the startle reflex. For an animal with tinnitus, it is expected that if the background sound in which the gap is embedded is qualitatively similar to the animal’s tinnitus, then tinnitus will ‘fill in’ the gap resulting in an impaired ability of the silent gap to inhibit the startle reflex. By comparing the ability of silent gaps in continuous background sounds of varying frequency and bandwidth to inhibit the startle reflex, the paradigm has been used to determine the pitch of an animal’s tinnitus. Using this paradigm, tinnitus has been assessed following exposure to salicylate as well as noise in a variety of species including rats, mice, guinea pigs, and hamsters (15, 44–47).

GPIAS has quickly become the most widely used tinnitus behavioral paradigm because it carries a number of advantages over the other previously reported tinnitus paradigms. It requires no behavioral training, no food or water deprivation, can assess tinnitus pitch, and allows for high-throughput screening for tinnitus. Because, there is no training involved, it can also be used to monitor animals for tinnitus repeatedly over long durations. In Turner’s original publication, group data was presented since baseline GPIAS measures were not completed prior to tinnitus induction via unilateral noise exposure (15). However, by collecting baseline and post-tinnitus induction GPIAS measures, the paradigm can be used to identify individual animals with tinnitus allowing for animals to be separated into tinnitus-positive and tinnitus-negative groups (48, 49).

However, despite these advantages, a number of concerns have recently been raised regarding the GPIAS paradigm and its use for screening animals for tinnitus. One concern is the discrepancy in tinnitus pitch reported in animals using the GPIAS paradigm following induction of tinnitus via exposure to high-frequency noise. Some studies, including the original Turner study, have reported the tinnitus pitch to fall below the noise exposure frequency (15, 50), while others report the tinnitus pitch to fall above the noise exposure frequency (44, 48, 51–53). Equally interesting is the finding that immediately following noise exposure, tinnitus has been found to occur across a wide range of frequencies but then becomes specific to a limited frequency band over the following weeks (54). In contrast, the tinnitus pitch in human subjects exposed to loud sound most frequently occurs at or above the noise exposure frequency (28, 29).

Another issue is the effect of hearing loss following exposure to noise or ototoxic drugs on the startle reflex amplitude used to assess pre-pulse inhibition. Hearing loss can potentially affect the outcome of GPIAS screening in a number ways: by interfering with audibility of the background sound in which the silent gaps are imbedded or by altering the amplitude of the startle reflex to the startle stimulus alone (no-gap condition). Previous studies in both rodents (55) and humans (56) have demonstrated that hearing loss alone, induced by sodium salicylate exposure, can interfere in detection of gaps in low-level continuous noise. This issue has been addressed in the GPIAS paradigm by using intensities of background sounds (60 dB SPL) shown to be resilient to the effects of hearing loss, and by carrying out noise-burst pre-pulse detection measures. During noise-burst detection measures, a short duration noise-burst of the same intensity as the background sound used in GPIAS testing is presented prior to the startle-eliciting stimulus to serve as the pre-pulse cue. It is assumed that if the noise-burst reliably inhibits the startle reflex, then audibility of the background sound in which the silent gaps are embedded during GPIAS testing should not be an issue.

In addition to the potential confounding effects of hearing loss on audibility of the background sound, another issue is that unilateral noise exposure can reduce the amplitude of the startle reflex during startle-alone (no-gap) trials (2, 44, 47, 50). In one study using rats, unilateral noise exposure resulted in a 57% reduction in the startle amplitude during startle-alone trials (2), while in another study using mice, unilateral noise exposure resulted in a 52% reduction of the acoustic startle reflex even after hearing thresholds recovered to pre-noise exposure levels (44). Alterations in startle reactivity pose a number of issues. First, as the dependent measure in the GPIAS paradigm, a robust startle reflex is needed in order to observe its inhibition. If animals fail to startle following manipulations to induce tinnitus, they will need to be excluded from further analysis, a practice reported in some previous studies using this paradigm (2, 50). Exclusion of animals from analysis not only reduces the high-throughput nature of the paradigm but may also result in the exclusion of animals that actually have tinnitus, but cannot be tested due to the absence of a robust startle reflex.

**Figure 4:**
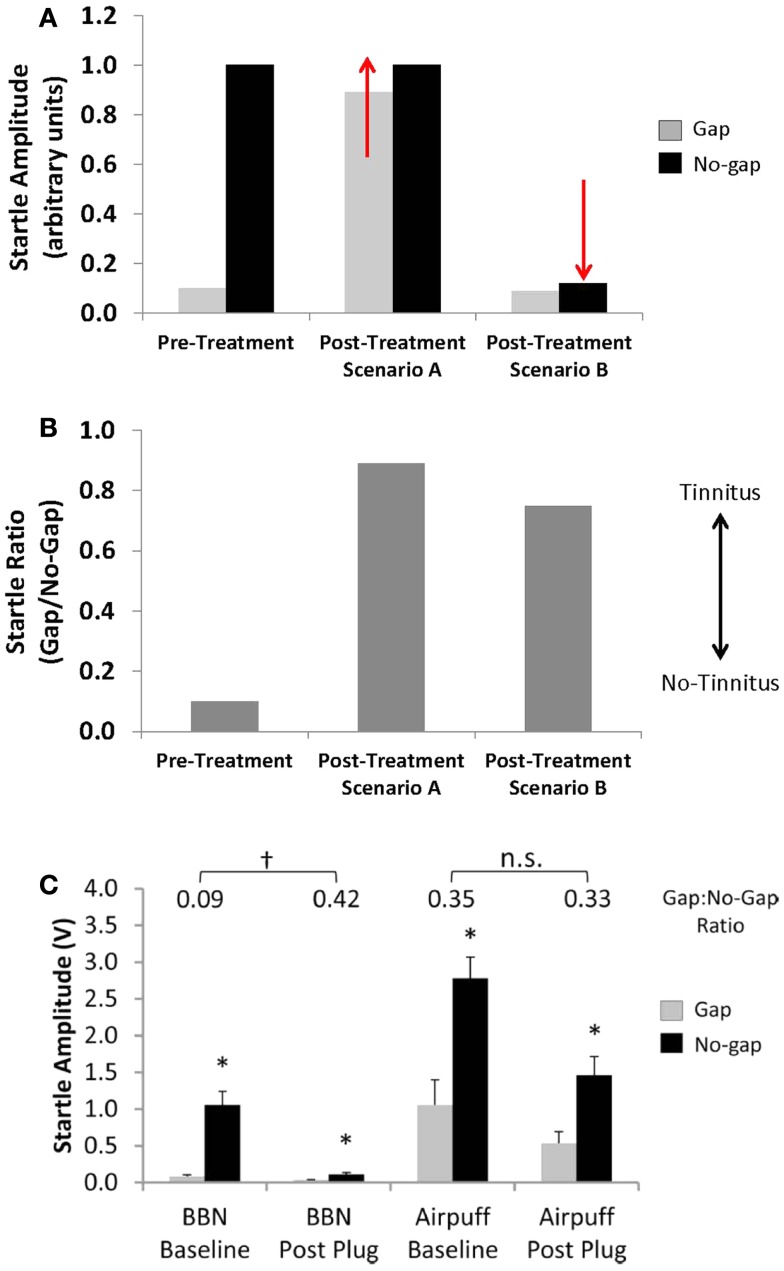
**Effects of alterations in startle magnitude on gap pre-pulse inhibition of the acoustic startle reflex (GPIAS) interpretation**. **(A,B)** A change in startle amplitude for either gap trials (Scenario A, gap-startle amplitude increases) or no-gap trials (Scenario B, no-gap-startle amplitude decreases) can result in changes in the GPIAS-Startle ratio indicative of tinnitus. **(C)** A decrease in no-gap trial startle amplitude, similar to the schematic of Scenario B, following temporary unilateral conductive hearing loss via an earplug has been shown to result in a false-positive screening for tinnitus in rats using the GPIAS paradigm. The false-positive screening for tinnitus could be eliminated by replacing the acoustic startle stimulus with a multi-modal airpuff startle stimulus which was more resilient to the effects of unilateral hearing loss on startle reflex amplitude (from Ref. (2), with permission; *indicates significant difference between startle amplitudes in gap versus no-gap trials; ^†^indicates significant differences between startle ratio values between baseline and post earplug measures; n.s. indicates no significant difference).

Second, alterations in baseline startle magnitude (i.e., no-gap trials) can potentially confound the interpretation of pre-pulse inhibition measures for both rodents and human subjects (2, 57). Given that GPIAS is calculated as a ratio between the startle amplitude in no-gap versus gap trials, a change in either parameter can result in a change in the GPIAS ratio. Traditionally, it was assumed that a change in the GPIAS ratio indicative of tinnitus was the result of an *increase* in the startle amplitude during gap trials if animals failed to detect the silent gap due to tinnitus “filling-in” the gap (Figures 4A,B; Scenario A). However, a change in the GPAIS ratio indicative of tinnitus can also occur if the no-gap-startle amplitude *decreases*, similar to what is seen following unilateral noise exposure (Figures 4A,B; Scenario B). Moreover, unilateral conductive hearing loss via an earplug has been shown to result in a false-positive screening for tinnitus in rats as a result of a reduction in startle magnitude during no-gap trials (Figure 4C) (2). Importantly, the false-positive screening for tinnitus could be eliminated by replacing the acoustic stimulus with a multi-modal airpuff stimulus (acoustic and somatosensory stimulation), which was more resilient to the effects of hearing loss (Figure 4C). Any changes in the no-gap-startle magnitude post-tinnitus induction need to be accounted for when using the GPIAS ratio as an indicator for tinnitus in order to ensure that GPIAS ratio changes are truly reflective of impaired detection of silent gaps, and not simply due to hearing loss (2). Close inspection of raw startle amplitudes before and after tinnitus induction, as well as controlling for methodological issues such as stimulus parameters and animal handling is strongly recommended when using and interpreting behavioral measures using the GPIAS paradigm (58).

**Figure 5:**
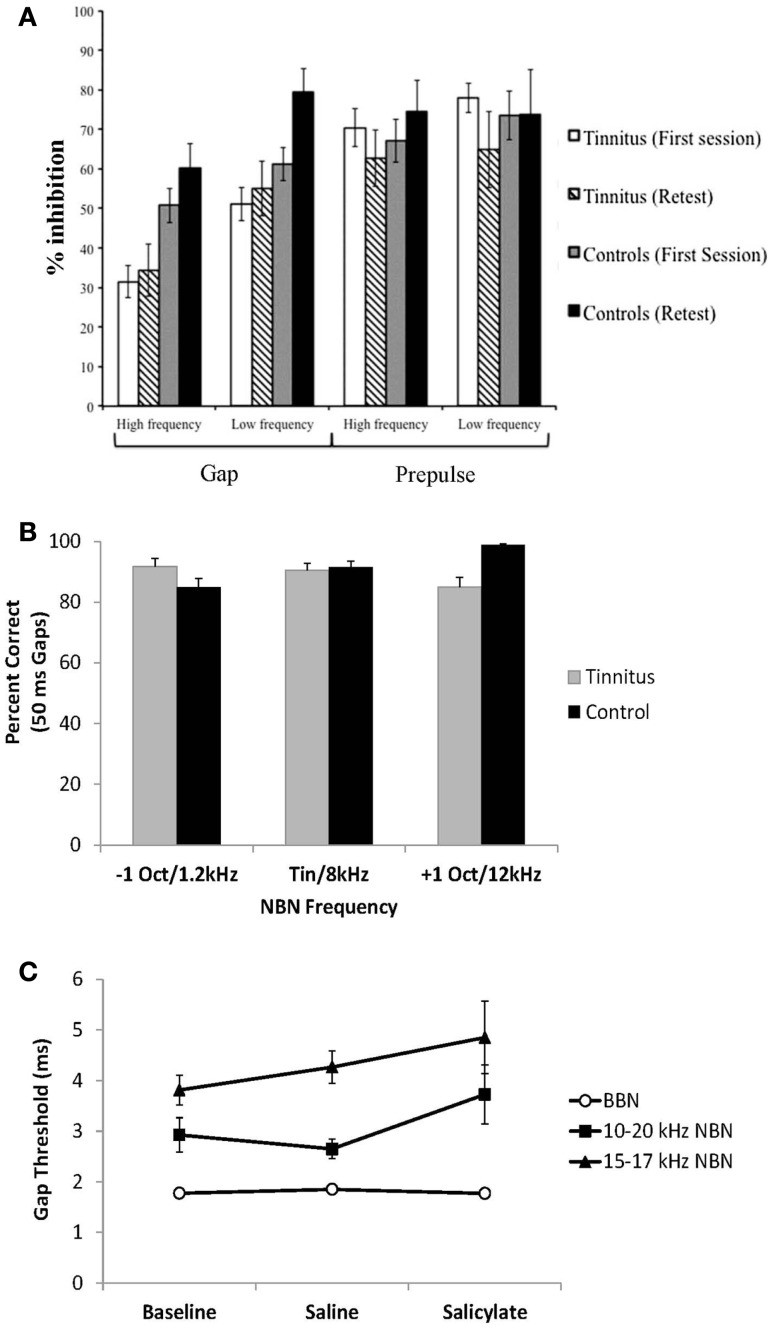
**Recent studies addressing the “tinnitus gap filling” hypothesis**. **(A)** Gap pre-pulse inhibition of the acoustic startle reflex measures in human tinnitus and control subjects. Tinnitus subjects demonstrate impaired gap detection (lower inhibition) for both low and high-frequency background sounds compared to control subjects [from Ref. (60), with permission]. **(B)** Subjective gap detection ability assessed in human tinnitus and control subjects. Both control and tinnitus subjects had no difficulty detecting 50 ms gaps presented in noise at 15 dB SPL. Narrow-band noises were presented 1 octave above, below or at the matched tinnitus pitch for tinnitus subjects and at 1.2, 8, and 12 kHz for control subjects without tinnitus [data from Ref. (59)]. **(C)** Gap detection assessed in rats trained on a go/no-go operant gap detection task to identify silent gaps embedded in continuous broad-band noise (BBN) and 10–20 or 15–17 kHz narrow-band noise (NBN) presented at 60 dB SPL. Salicylate had no significant effect on gap detection, as gap duration thresholds remained below 6 ms [data from Ref. (62)].

In addition to the potential effects of hearing loss on GPIAS measures, another issue is whether the hypothesis that tinnitus “fills in the gap” is accurate. Recent studies in both humans (59, 60) and rodents (61, 62) have addressed this issue. In one GPIAS study, human patients with high-frequency tinnitus were found to have impaired gap detection for gaps presented in both low and high-frequency background stimuli compared to control subjects (60) (Figure 5A). Since these patients with high-frequency hearing loss also had high-frequency tinnitus, GPIAS should only be impaired at high frequencies, not at low frequencies. Because their tinnitus patients were found to have significantly less inhibition of the startle response irrespective of frequency, the authors suggest that the impaired gap detection may be reflective of a more general cortical processing disorder rather than tinnitus “filling in the gaps.”

In another study, gap detection ability was assessed in human tinnitus patients by asking whether they could perceive 50 ms gaps in 15 dB SL background sounds presented either at their tinnitus pitch, or one octave above and below their tinnitus pitch (59) (Figure 5B). Both control and tinnitus subjects had no difficulty detecting the silent gaps irrespective of background frequency (i.e., tinnitus did not “fill in” the silent gaps at frequencies above, below, or at the tinnitus pitch). It is important to note, however, that a direct comparison in gap detection cannot be made between the tinnitus and no-tinnitus subjects in this study because the two groups were tested with different background stimuli (control subjects did not have tinnitus and therefore could not have background stimuli matched to their tinnitus pitch). Instead, by comparing within the group of tinnitus subjects across frequency, the data demonstrated that tinnitus did not “fill in” or interfere with detection of silent gaps in background sounds at the tinnitus frequency or at frequencies one octave above and below the matched tinnitus frequency (Figure 5B, compare gray bars for each frequency). In a similar study, Boyen et al. (63) recently found that human tinnitus patients had similar gap detection thresholds compared to a matched non-tinnitus control group even when the test frequency matched the patient’s tinnitus frequency (63).

In addition to human studies, a number of animal studies have also investigated the “tinnitus filling the gap” hypothesis. Hickox and Liberman (61) demonstrated that gap detection deficits in noise-exposed rodents tested with the GPIAS paradigm are dependent on the interval between the silent gap and the startle-eliciting stimulus (43). Noise-exposed animals demonstrated GPIAS deficits only when the silent gap was placed immediately before the startle stimulus, but not when it was placed 80 ms before the startle stimulus. The authors concluded that these results are inconsistent with the “tinnitus filling the gap” hypothesis, as gap detection deficits from tinnitus filling in the gap should be seen irrespective of where the silent gap is placed (i.e., at any placement where GPIAS would normally be observed) (61).

Lastly, gap detection has also been assessed in rodents trained on a go/no-go operant gap detection task to determine the threshold for silent gaps embedded in continuous background sounds (62) (Figure 5C). In this study, rats were treated with a dose of sodium salicylate known to reliably induce tinnitus (18). Following salicylate administration, gap detection thresholds were unchanged for gaps embedded in broad-band noise or narrow-band noises presented at 60 dB SPL (the same intensity background noise commonly used in the GPIAS paradigm). These results indicate that salicylate-induced tinnitus does not “fill in the silent gaps.” Taken together, the results suggest that tinnitus assessment with the GPIAS paradigm should be interpreted with considerable caution. Ultimately, the rationale for using GPIAS as a behavioral test for tinnitus in animals should be based on the ability of the paradigm to accurately assess tinnitus in human patients.

## Human Studies of Hyperacusis

Hyperacusis, defined as a hypersensitivity to moderate-intensity sounds or abnormal loudness perception (4–7), often co-occurs with tinnitus (6, 64–66). The prevalence of hyperacusis in the tinnitus population was estimated to be ~80% (8). The frequent co-occurrence of these two perceptual disorders suggests a common mechanism(s) of dysfunction (7, 64, 66), such as an increase in central gain following hearing loss (67–69). Given the high rate of overlap between these two disorders, it is important to discuss hyperacusis when assessing models of tinnitus (24, 66, 70).

To clarify, hyperacusis is distinct from loudness recruitment, the abnormally rapid growth in perceived loudness with increasing intensity, in that it does not necessarily coincide with threshold elevation and hair cell damage, but does feature reduced loudness discomfort levels (6, 61, 71). In addition, hyperacusis is not sound-specific and anxiety can aggravate symptoms (5, 71).

Generally, hyperacusis is measured using loudness rating scales because the primary feature of this auditory perceptual disorder is a reduced tolerance for moderate-level and intense sounds (6, 7, 47). To assess a listener’s sensitivity to sounds, participants are typically instructed to rate sounds according to pre-determined categories of loudness (such as 1 for quiet and 10 for painfully loud) (72, 73). Although these subjective rating scales are useful measures in humans, these methods are impossible to use in animals because they require a listener’s ability to follow instructions and adjust, or rate, stimuli accordingly (74). Therefore, researchers have turned to objective behavioral measures of loudness perception, such as the amplitude of the ASR and operant conditioning techniques using reaction time (RT) measures, as methods for estimating perceived loudness in both animals and humans.

## Animal Models of Hyperacusis

### Acoustic startle reflex paradigm

**Figure 6:**
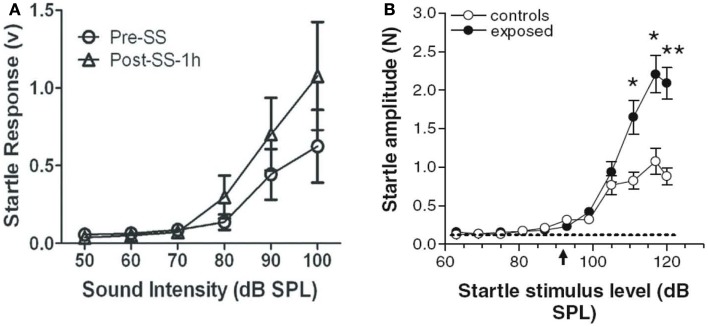
**Animal models of hyperacusis using the acoustic startle reflex (ASR) paradigm**. **(A)** Mean ASR amplitudes in rats pre-sodium salicylate injection (open circles) and 1 h post-sodium salicylate injection (250 mg/kg i.p.) (open triangles). Startle amplitudes increased significantly at high sound intensities 1 h after salicylate injection [from Ref. (77), with permission]. **(B)** Mean ASR amplitudes in hamsters following noise exposure (10 kHz, 115 dB SPL, 4 h) (black circles) or in unexposed control hamsters (open circles). Startle amplitudes were significantly higher at high sound intensities in noise-exposed hamsters than in unexposed hamsters, suggesting increased loudness sensitivity in noise-exposed animals [from Ref. (47), with permission].

The acoustic startle reflex (ASR) paradigm has been used by a number of researchers to assess animals for age-related (75), drug-induced (76, 77), and noise-induced (47, 61, 78, 79) hyperacusis. According to these studies, an animal is thought to have hyperacusis if the amplitude of its startle reflex, a short-latency, robust motoric response (80, 81), increases after some manipulation, such as an injection of sodium salicylate or a noise exposure (47, 77) (Figures 6A,B). Like the GPIAS reflex paradigm for assessing tinnitus, the ASR paradigm is an efficient, high-throughput behavioral method because it requires no training or learning. In addition, the ASR paradigm is attractive because it does not require any food or water restriction or the use of electric shock.

However, the ASR paradigm can be problematic for several reasons. For one, it is difficult to discriminate hyperacusis from generalized, non-auditory-specific hyperactivity with ASR alone (23, 82). Secondly, enhanced ASRs have been reported to be more predictive of tinnitus rather than hyperacusis in humans, questioning ASR’s usefulness in assessing hyperacusis in animals (83). In addition, it is unclear how the ASR paradigm can be used to differentiate hyperacusis from loudness recruitment. Lastly, changes in the ASR tend to occur only for high-intensity sounds (90 dB SPL or higher), which suggests that this method may not be sensitive to changes in loudness perception at moderate sound levels, one of the defining features of hyperacusis (78, 84). Although these potential limitations need to be addressed, the ASR paradigm appears, for now, to be an effective tool for estimating loudness perception, hyperacusis, or sound-evoked hyper-reactivity in animals at intensities greater than 80 dB SPL.

### Operant conditioning methods with reaction time measures

Since RT is a reliable surrogate of perceived loudness (72, 85–89), and loudness growth functions appear to be the perceptual correlate of hyperacusis in humans (7), researchers have obtained RT measures using operant conditioning techniques to assess loudness perception and hyperacusis in both animals and humans (74).

**Figure 7:**
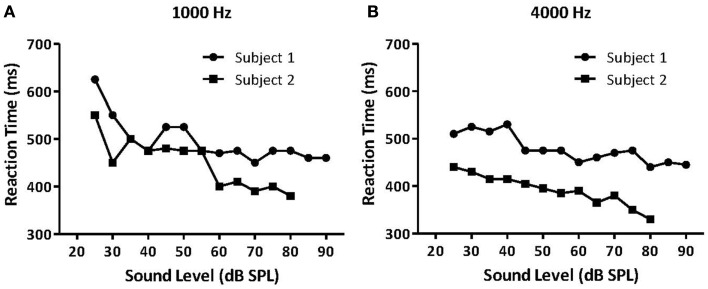
**Median reaction times (RT) for a normal-hearing human listener (Subject 1, black circles) and a listener with reduced loudness tolerance (Subject 2, black squares)**. Median RTs for both listeners for **(A)** 1000 Hz tones and **(B)** 4000 Hz tones presented at various intensities. Subject 2 had significantly faster RTs to moderate and high-intensity sounds than Subject 1, suggesting that the participant with hyperacusis perceived these sounds as louder than the normal-hearing participant, and that RT can potentially measure hyperacusis in humans [modified from Ref. (74)].

The first study to examine hyperacusis in animals and humans using RT measures was Lauer and Dooling (74). They measured RTs in both normal-hearing canaries and an inbred strain of canary with a permanent hereditary high-frequency hearing loss, the Belgian Waterslager (BWS). They hypothesized that animals with hyperacusis should show faster than normal RTs because sound stimuli are perceived as being louder than in normal-hearing animals. In accordance with that hypothesis, they found that BWS canaries had near-normal RTs at lower sound levels but much faster RTs than normal canaries at higher sound levels (74). Importantly, Lauer and Dooling (74) verified these methods by testing two humans, one with hyperacusis and one without, using the same behavioral methods as in the canary experiment (Figures 7A,B).

**Figure 8:**
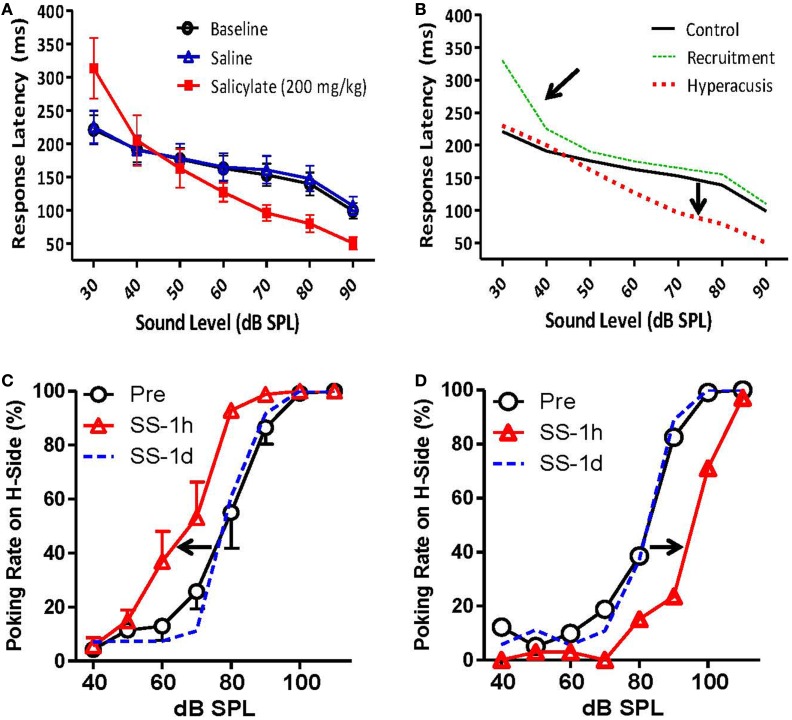
**Animal models of hyperacusis using operant conditioning procedures**. **(A)** Mean reaction times (RT) in rats to broad-band noise bursts for baseline (open circles), saline (open triangles), and salicylate (200 mg/kg i.p.) (filled squares) conditions. RTs decreased significantly for 70, 80, and 90 dB SPL noise bursts following the injection of salicylate, suggesting an increased sensitivity to loud sounds [modified from Ref. (84)]. **(B)** Hypothetical data indicating hyperacusis (red dashes) and loudness recruitment (green dots) using the go/no-go reaction time model. Top arrow indicates steep loudness growth function seen in listeners with loudness recruitment. Bottom arrow shows faster reaction times for moderate to intense sounds, indicative of increased loudness perception in listeners with hyperacusis. **(C,D)** Mean number of responses to the 100-dB SPL feeder pre-salicylate (open circles) and 1 h post-salicylate (250 mg/kg i.p.) (red triangles) for individual rats. **(C)** The number of responses to the high-intensity (100 dB SPL) feeder increased significantly following salicylate administration (arrow indicates shift to the left), suggesting increased loudness sensitivity and hyperacusis. **(D)** The number of responses to the high-intensity (100 dB SPL) feeder decreased significantly following salicylate administration (arrow shows shift to the right), indicative of loudness recruitment [from Ref. (90), with permission].

Recently, Chen et al. (84) measured RTs in rats both before and after a large dose of sodium salicylate that is known to induce hearing loss and tinnitus in animals (18). Briefly, the rats were trained to detect broad-band noise bursts in an otherwise quiet chamber using a *Go/no-go operant conditioning paradigm*. RT measures were taken from the onset of the noise burst to the time the rat made a response, and only RTs for “hits” (when the animal correctly detected the stimulus) were included in the analysis (84). Chen et al. (84) found that RTs post-salicylate were faster than pre-salicylate for noise bursts 70 dB SPL or greater but were the same or even slower to respond to noise bursts at 50 dB SPL or less (84) (Figure 8A). Importantly, this go/no-go paradigm can differentiate hyperacusis from loudness recruitment. Hyperacusis is evidenced by faster RTs than normal for moderate to intense sounds, while loudness recruitment associated with hearing loss is characterized by slower RTs for low-level sounds with a rapid loudness growth function and normal RTs to moderate to intense sounds (Figure 8B).

In another operant conditioning paradigm, Sun et al. (91) tested adult rats with tympanic membrane (TM) damage as pups, and Zhang et al. (90) tested rats before and after a large dose of sodium salicylate for hyperacusis in a *two-alternative forced choice (2AFC) operant conditioning task*. In both experiments, animals were trained to make a response to the right nose-poke hole when they heard a 100-dB SPL sound and to the left side when they heard a 60-dB SPL sound. After the rats were correctly identifying these two stimuli, probe stimuli were included from 40 to 110 dB SPL that were always reinforced with a food pellet regardless of which side the animals chose (90, 91). They found that rats with TM damage as pups, and 40% of rats given salicylate, labeled mid-intensity stimuli (70 and 80 dB SPL) as 100 dB SPL more often than control rats, suggesting that rats with prior TM damage and rats given salicylate perceived these stimuli as louder than normal (Figure 8C). Interestingly, several rats given salicylate appeared to experience loudness recruitment following an injection of salicylate. Instead of responding more frequently to the 100-dB SPL feeder, these rats responded more frequently to the 60-dB SPL feeder, indicating a loss in sensitivity at lower sound levels and an abnormally steep loudness growth function (Figure 8D).

Although the above-mentioned go/no-go and 2AFC operant conditioning tasks deliver reliable and stable measures in animals and humans, these tasks require weeks of animal behavioral training and the animals need to be food or water-restricted in order to perform in the task. Nevertheless, the RT paradigm has been extensively validated as a measure of loudness (72, 85–89) and it may provide the most reliable and sensitive estimates of loudness perception in *both* animals and humans.

## Conclusion

A reliable behavioral paradigm is vital for identifying the underlying neural mechanisms and potential therapeutic treatments for tinnitus and hyperacusis. Ultimately, any behavioral model of hyperacusis or tinnitus should closely mirror what we know about these disorders in the human population. Given the range of behavioral training techniques and methodologies used to assess tinnitus and hyperacusis, there are a number of important characteristics to consider when evaluating their utility and consistency with human data. An ideal behavioral paradigm should be able to identify the presence of the condition in individual animals, allow for long-term testing of animals for studies of chronic tinnitus and hyperacusis, should be resilient to any secondary effects of the induction method including hearing loss, and would have a relatively short training and testing time. Additionally, an ideal model of tinnitus would also allow for measurements of tinnitus pitch and loudness, while an ideal model of hyperacusis should allow for the differentiation between the presence of hyperacusis and loudness recruitment. Clearly, the advancements in animal models for tinnitus and hyperacusis have come a long way and will continue to play an important role in revealing the underlying mechanisms and treatments for tinnitus and hyperacusis.

## Author Contributions

Sarah H. Hayes, Kelly E. Radziwon, Daniel J. Stolzberg, and Richard J. Salvi wrote the manuscript and approved the final version.

## Conflict of Interest Statement

The authors declare that the research was conducted in the absence of any commercial or financial relationships that could be construed as a potential conflict of interest.
